# Mental health trajectory throughout high school football career: a four-year prospective cohort study

**DOI:** 10.3389/fpsyt.2025.1723687

**Published:** 2025-12-03

**Authors:** Claire V. Buddenbaum, Grace O. Recht, Sage H. Sweeney, Jesse A. Steinfeldt, Gage Ellis, Taylor R. Zuidema, Kyle A. Kercher, Jeffrey J. Bazarian, Sharlene D. Newman, Keisuke Kawata

**Affiliations:** 1 Department of Kinesiology, Indiana University School of Public Health-Bloomington, Bloomington, IN, United States; 2 Department of Applied Psychology in Education & Research Methodologies, School of Education, Indiana University, Bloomington, IN, United States; 3 Program in Neuroscience, The College of Arts and Sciences, Indiana University, Bloomington, IN, United States; 4 Department of Emergency Medicine, University of Rochester School of Medicine and Dentistry, Rochester, NY, United States; 5 Alabama Life Research Institute, University of Alabama, Tuscaloosa, AL, United States; 6 Department of Pediatrics, Indiana University School of Medicine, Indianapolis, IN, United States

**Keywords:** brain injury, concussion, subconcussive head impacts, depression, anxiety, football

## Abstract

**Introduction:**

Repetitive head impacts may contribute to long-term neurological disorders, including chronic traumatic encephalopathy (CTE), and mental health decline often precedes cognitive symptoms. Adolescent athletes are especially vulnerable, yet prospective data on mental health trajectory across high school athletic career are limited.

**Aim:**

To examine the mental health changes of high school football players across multiple seasons of participation.

**Methods:**

This prospective cohort study included 6 high schools across southern Indiana for 4 consecutive seasons from July 2021 to February 2025. Participants included male high school football players and noncontact athletes (ages 13-18) in tennis, cross-country, and swimming. Sensor installed mouthguards were utilized to measure head impacts and surveys were conducted pre and post season to assess anxiety and depressive symptoms.

**Results:**

A total of 371 adolescent athletes participated in this longitudinal study, including 275 high school football players (mean [SD] age, 15.3 [1.2] years) and 96 non-contact control athletes (mean [SD] age, 15.9 [1.2] years), with varying lengths of participation across 4 years of study. Depression and anxiety symptoms remained consistent across 1 season (pre vs. postseason) in both groups, as illustrated by non-significant group by time interaction in PHQ-9 [b=-0.11, 95%CI(-0.98, 0.76), p=0.814] or GAD-7 [b=0.09, 95%CI(-0.62, 0.79), p=0.806]. The same pattern was observed for those who participated in 2 consecutive seasons [PHQ-9 b=-0.01, 95%CI (-0.96, 0.98), p=0.980; GAD-7 b=0.42, 95%CI(-0.43, 1.27), p=0.33]. However, among those with 3 consecutive years of participation, there was significant seasons (pre vs. post) by year (1^st^ vs. 3^rd^ year) interaction in depression symptoms in the football group [PHQ-9: b=1.50, 95%CI (0.32, 2.66), p=0.015], a pattern not observed in the control group.

**Discussion:**

Our data suggest that mental health wellbeing remain consistent across 1 to 2 years of high school sports participation, regardless of head impact exposure or types of sports they play (football or non-contact sports). However, psychological burden may accumulate over multiple years of football participation.

## Introduction

Over the past decade, the potential link between repetitive head impacts and chronic traumatic encephalopathy (CTE) has become a central topic in neurotrauma research and public discussion. This concern is particularly prominent in American football, where players may endure hundreds of head impacts each season, including both concussive and subconcussive head impacts (1, 2). While a concussion elicits a range of physical, mental, and cognitive symptoms, even asymptomatic head impacts can leave a biological footprint, including elevated brain-injury blood biomarkers (3–5), impairments in sensorimotor function (3, 6), and axonal microstructural damage (7, 8). While prospective longitudinal data remain scarce, retrospective research suggests that neurobiological consequences may accumulate over years of head impact exposure (9, 10). Historically, CTE research has focused on middle-aged and older adults; however, emerging evidence indicates that neurodegeneration may begin as early as in adolescence. Autopsy studies of young adults and teenagers exposed to repetitive head impacts (RHI) have identified early signs of tau tangles and neuroinflammation (11). As mental health changes often precede cognitive decline in CTE (10, 11), tracking psychological wellbeing throughout a high school football career is critical for understanding early disease progression.

The impact of concussion on mental health has been extensively studied. For example, a large-scale cohort study by Ledoux et al. (12) reported that 152, 321 children and youths with a concussion (median age: 13 years) had a 1.4-fold higher incidences of mental health disorders (e.g., anxiety, neurotic, mood disorders), self-harm, and psychiatric hospitalization compared to orthopedic injury controls (12). Furthermore, teenagers who sustain concussion are at a 3-fold increased risk of developing depression within 6 months of injury (13), and suicide attempts in high schoolers are higher among those with more than 2 concussion history (14). Such findings highlight the long-lasting mental health effects of concussion on the developing brain.

Beyond the risks of concussion, contact sport athletes experience hundreds of subconcussive head impacts, defined as impacts that do not produce overt symptoms. Using a decade of head impact sensor data, Montenigro et al. (15) demonstrated that exceeding a cumulative threshold of 1, 801 head impacts is associated with a linear, dose-dependent increase in the risk of developing depression among former high school and college football players. Moreover, a recent study of middle-aged adults with at least 10 years of contact or non-contact sport participation revealed elevated symptoms of post-traumatic stress disorder (PTSD), depression, and attention-deficit/hyperactivity disorder (ADHD) in the contact sports group compared to the non-contact controls (16). The contact group was 2.25 times more likely to receive mental health disorder diagnoses and 1.29 times more likely to use related medications (16). However, contradictory findings exist. For example, Iverson et al. (17) reported no differences in lifetime diagnosis of depression, anxiety, panic disorders, or suicidal ideation between former high school football players and age-matched controls (17). While these studies offer valuable insights into the relationship between head impacts and mental health, many are limited by cross-sectional designs, proxy measures of cumulative head impact exposure, and reliance on diagnostic categories rather than detailed symptom scores.

These limitations underscore the need for prospective studies to clarify the temporal relationship between RHI and mental health outcomes. Our previous pilot study by Kercher et al. (18) took an initial step toward addressing this gap, reporting no significant changes in depression or anxiety scores over a single high school football season, nor any correlation between mental health outcomes and head impact kinematics measured via sensor-installed mouthguards. However, a key question remains: does mental health remain stable across multiple seasons, or does it decline with prolonged exposure to RHI?

This multisite, longitudinal cohort study spanning 4 years aimed to characterize the trajectory of mental health changes among high school football players and examine the potential modulatory role of RHI on mental health outcomes. The study was guided by two primary objectives: (1) to replicate our pilot findings by assessing mental health wellbeing over a single football season and (2) to evaluate whether the magnitude of pre- to post-season changes in depression and anxiety symptoms differs following two or three consecutive years of football participation. We hypothesized that depression and anxiety scores would remain stable across a single football season, with no significant differences between football players and non-contact sport controls. Furthermore, multiple seasons of football participation would be associated with a progressive decline in mental health among adolescent football players.

## Materials and methods

### Participants

This multisite, longitudinal cohort study, spanned across 4 academic school years, from July 2021 to February 2025. It included 373 adolescent male athletes, 275 football players and 98 noncontact sport athletes from across 6 high schools in the Midwest. Inclusion criteria consisted of being an active football or noncontact athlete (swimming, cross-country, and tennis), between the ages of 13 and 18, and willing to wear a sensor-installed mouthguard. Exclusion criteria included a history of severe traumatic brain injury (TBI) and moderate TBI/concussion with symptoms present in the past 6 months. All participants and legal guardians signed consent forms prior to each year of participation. Approval was obtained from the district school board and school stakeholders, and Indiana University Institutional Review Board approved the study protocol (1904461516).

### Study procedures

During preseason baseline, participants completed an online questionnaire that captured demographic information, contact sport history, football-specific context, concussion history, and mental health diagnosis. Prior to each season, football participants were fitted with a sensor-installed mouthguards (Prevent Biometrics, Inc.) and wore them for all practices, scrimmages, and games until the conclusion of their season. Mental health questionnaires were administered in-person, in the presence of research staff, at both pre- and post-season each year for both football and control athletes.

### Measures

#### Depressive symptoms

The Patient Health Questionnaire-9 (PHQ-9) was used to assess self-reported depressive symptoms. The PHQ-9 consists of 9 items rated on a 4-point Likert scale (0 to 3), where 0 indicates “not at all”, and 3 indicates experiencing the symptom “nearly every day”. A total score ranges from 0 to 27, and higher scores represent worse depressive symptom (19). Among adolescents, the PHQ-9 has demonstrated strong internal consistency with a Chronbach’s alpha coefficient of 0.82, alongside a sensitivity of 89.5% and a specificity of 77.5% for detecting youth with major depressive disorder (20).

#### Anxiety symptoms

The General Anxiety Disorder-7 (GAD-7) is a self-administered questionnaire that assesses worry and anxiety symptoms. The GAD-7 consists of 7 questions with a 4-point Likert scale (0 to 3), where 0 indicates “not at all”, and 3 indicates experiencing the symptom “nearly every day”. Total scores range from 0 to 21 with higher scores reflecting greater anxiety severity (21). In adolescents, the GAD-7 has shown a strong reliability (Cronbach’s alpha=0.91) and adequate construct validity (22).

#### Head impacts

This study employed the Prevent Biometrics Instrumented Mouthguard (IMM) system, which integrates a triaxial accelerometer (ADXL372) and gyroscope (BMG250) to capture six degrees of freedom in head kinematics, including both linear and rotational accelerations. When acceleration along any axis exceeds a preset threshold (5–15 g), the device initiates data recording. Each event is processed by the mouthguard’s onboard firmware and transmitted via Bluetooth to a mobile application, which subsequently uploads the encrypted data to a secure, cloud-based server. The system samples at 3.2 kHz and records 50 ms of data per event. The mouthguard’s internal memory can store up to approximately 460 impacts and contains sensors that verify proper fit and use during athletic activity. For the present analyses, only head impacts with peak linear acceleration greater than 10 g were retained to minimize inclusion of non-impact movements such as jumping or running (23, 24). Research equipment managers were onsite during every practice and game to ensure the deployment of mouthguards, sanitation, charging of mouthguards, and synching impact data. Given the significant correlations (r<0.90, p<0.0001) among head impact counts, cumulative peak linear acceleration (*g*), and cumulative peak rotational acceleration (krad/s^2^), we chose head impact counts as our predictor in the analyses. The film validation of a randomly selected 1, 785 head impacts resulted in 94% (n=1, 670) of agreement between mouthguard data and film analysis (25).

### Statistical analysis

Group differences (football or control) in demographic variables were assessed with two-tailed independent samples t-tests for continuous variables and chi-squares for categorical variables. To examine changes in mental health outcomes across one season, we conducted mixed-effects regression (MRM) models on the primary outcome of PHQ-9 and GAD-7 scores. The primary (fixed-effect) factor was timepoint (pre- vs. post-season), group (football vs. control), and the group-by-time-interaction. Participants were treated as a random effect to account for individual differences. The model accounted for repeated measures from the same participant and included covariates of age, number of concussions, a binary variable representing if they had a mental health diagnosis (0=no diagnosis, 1=mental health diagnosis), BMI, and race. The secondary analysis focused on the football group, evaluating changes in PHQ-9 and GAD-7 scores across second or third consecutive seasons. Fixed effects included timepoint (pre- vs post-season) and season (first vs. second; first vs. third seasons), with the same covariates from the primary analysis. As part of a sensitivity analysis, identical MRMs were run for control athletes to determine whether observed trends were specific to football participation. Analysis for each model was summarized by providing a contrast estimate with its 95% confidence interval (CI) and p-value in the following format (b estimate [95% CI, low CI, high CI], p-value). The significance level for all tests was set to 0.05. *Post-hoc* analyses were conducted using marginal means to examine group differences when necessary. All analyses were conducted using R, version 4.4.2 (R Project for Statistical Computing) using packages nlme and emmeans.

## Results

### Demographics

**Figure 1:**
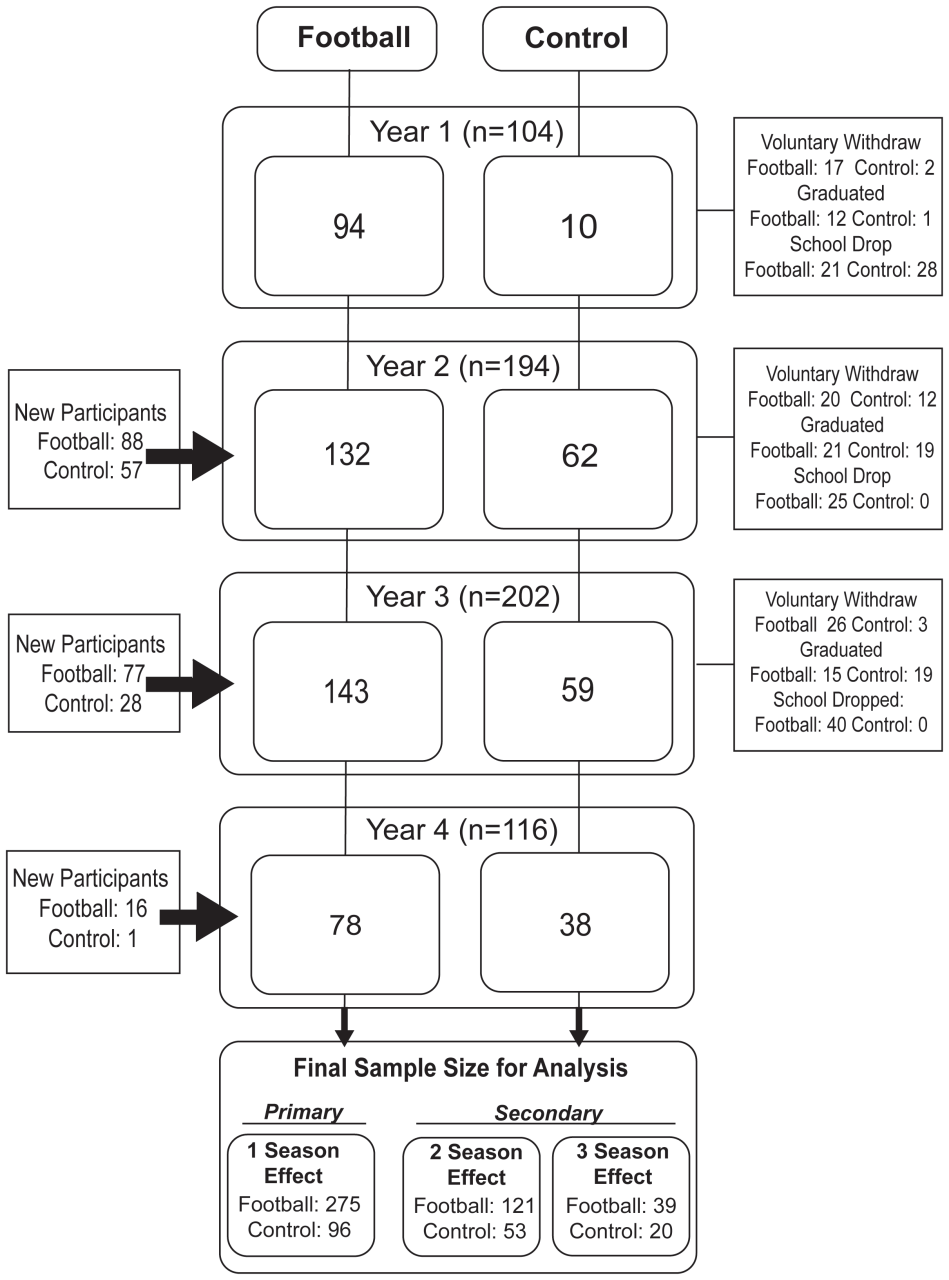
Study flow chart.

A total of 371 adolescent athletes participated in the study, including 275 high school football players (mean [SD] age, 15.3 [1.2] years) and 96 non-contact control athletes (mean [SD] age, 15.9 [1.2] years) throughout 4 years of study. Participants completed varied durations of the study (**Figure 1**). Sample sizes contributed to our analyses were: 1^st^ season (n=275 football, n=96 control), 2 consecutive seasons (n=121 football, n=53 control), and 3 consecutive seasons (n=39 football, n=20 control). Given the limited sample size participating in the 4 consecutive seasons, their data up to 3 seasons were included in the analyses. Football players were slightly younger, had a higher body mass index (BMI), and more racially diverse than the control athletes. See **Table 1** for demographics.

**Table 1:** Group demographics at time of initial enrollment.

Group	Football	Control	P-value
n	275	96	–
Sex (%)	275 (100)	96 (100)	–
Age, y	15.3 (1.2)	15.9 (1.2)	<0.001
Body Mass Index, *mean (SD)*	25.9 (6.0)	20.7 (3.0)	<0.001
No. of previous concussion			0.08
0, *n* (%)	219 (79.6)	86 (89.6)	–
1, *n* (%)	45 (16.4)	8 (8.3)	–
2, *n* (%)	9 (3.3)	1 (1.0)	–
3+, *n* (%)	0 (0.0)	1 (1.0)	–
Position, n (%)*			–
Lineman	98 (35.6)	–	–
Hybrid	73 (26.5)	–	–
Skill	93 (33.8)	–	–
Race, *n* (%)			0.03
White	229 (83.3)	85 (88.5)	–
Black/African American	24 (8.7)	1 (1.0)	–
Asian	4 (1.5)	5 (5.2)	–
American Indian or Alaska Native	2 (0.7)	0 (0.0)	–
Native Hawaiian or Pacific Islander	1 (0.4)	1 (1.0)	–
Multiracial	15 (5.4)	4 (4.2)	–
Ethnicity, *n* (%)			0.61
Not Latino/Hispanic	261 (94.9)	93 (96.9)	–
Latino/Hispanic	14 (5.1)	3 (3.1)	–
Mental Health Diagnosis, *n (%)*			0.56
Yes	43 (15.6)	12 (12.5)	–
No	232 (84.4)	84 (87.5)	–
Head Impact Frequency			
Year 1, *mean (SD)*	92.8 (110.8)	–	–
Year 2, *mean (SD)*	97.2 (148.4)	–	–
Year 3, *mean (SD)*	57.2 (77.0)	–	–
Year 4, *mean (SD)*	110.9 (154.6)	–	–

*Not equal to 100 due to missing positions. SD, standard deviation. y; year.

### Impact of 1 football season participation on mental health outcomes

**Figure 2:**
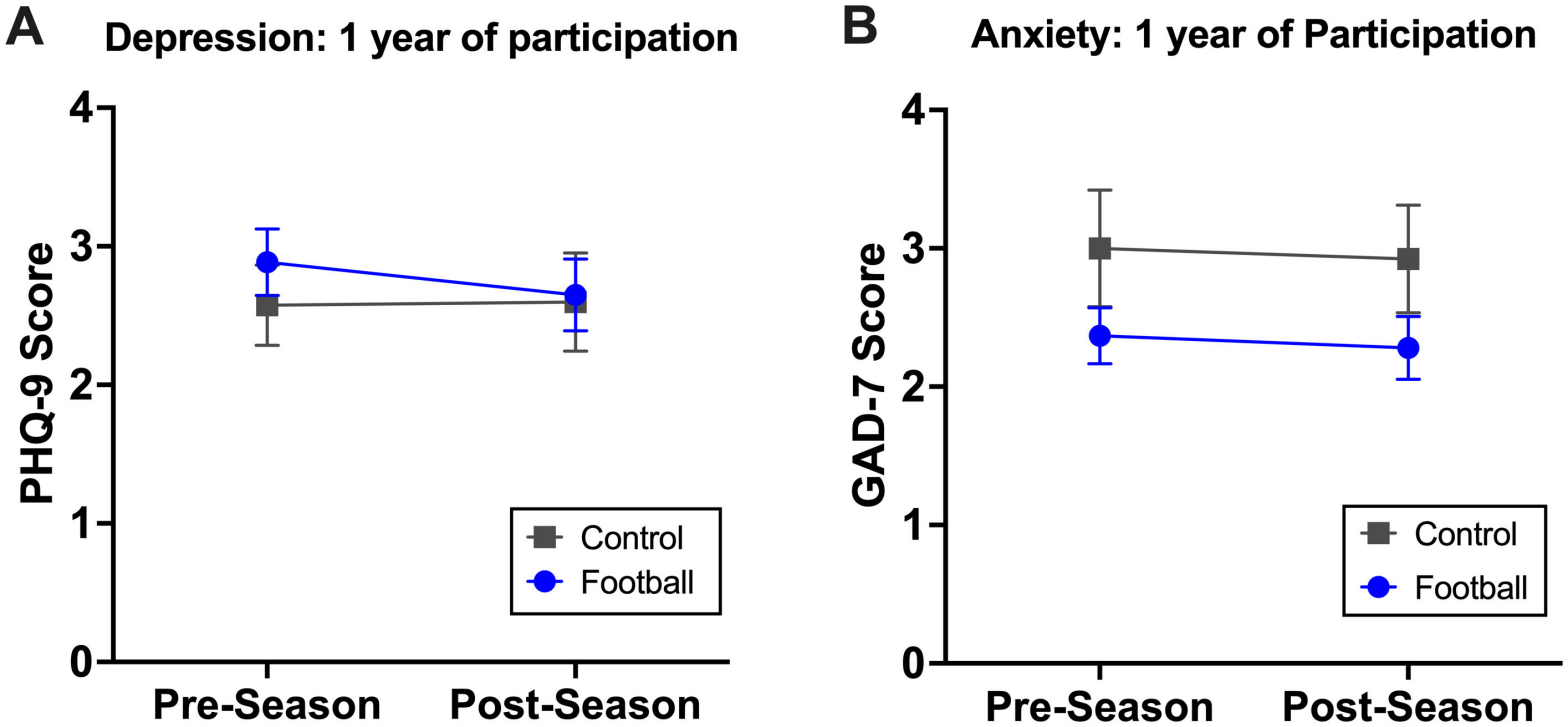
A single football season effects on depression and anxiety. Among 275 football players and 96 control athletes, there were no significant changes in depression **(A)** or anxiety **(B)** across one season.

There were no significant differences in depression and anxiety scores across one season (preseason vs. postseason) or between groups, as illustrated by no significant group-by-time interaction [PHQ-9, b=-0.10, 95%CI(-0.98, 0.76), p=0.814; GAD-7, b=0.09, 95%CI(-0.62, 0.79), p=0.806; **Figure 2]**. More specifically, football players exhibited 2.89±3.90 in PHQ-9 score (depression) and 2.37±3.22 in GAD-7 score (anxiety) at preseason, which were comparable to their postseason (depression 2.65±4.05; anxiety 2.28±3.49), as well as those of control athletes (preseason: depression 2.57±2.81, anxiety 3.00±4.09; postseason: depression 2.60±3.39, anxiety 2.92±3.74; **Supplementary Table 1**). Of note, a history of mental health diagnosis was significantly associated with elevated depression and anxiety scores regardless of groups or timepoints. Conversely, there was no association between mental health outcomes and cumulative head impacts during a season [PHQ-9: b=-0.003, 95%CI(-0.008, 0.002), p= 0.201; GAD-7: b=-0.002, 95%CI(-0.006, 0.002), p=0.332].

### Multi-season effects on mental health outcomes

**Figure 3:**
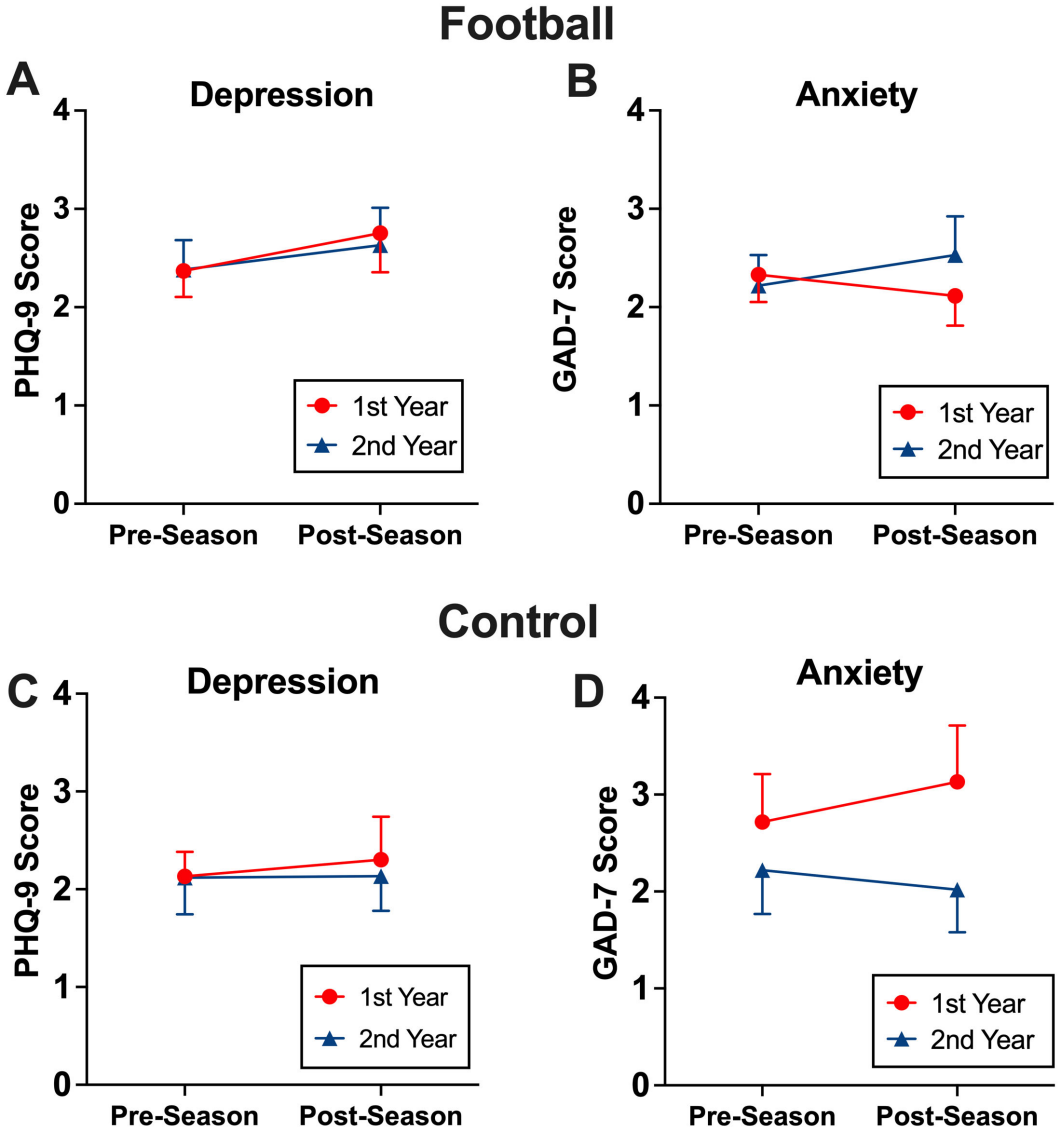
The effects of two consecutive season on mental health. Among those who participated two consecutive seasons, football players and control athletes maintained depression **(A, C)** and anxiety **(B, D)** symptoms after the 2^nd^ season similar to their 1^st^ season.

Mixed-effects regression models were used to examine associations between multiple seasons of football participation and alterations in mental health status. First, among football players participating in 2 consecutive seasons, there were no statistically significant differences in the pattern of mental health changes across 2 seasons, as evidenced by no significant season (pre vs. post) by year (1^st^ vs. 2^nd^ year participation) interaction [PHQ-9 b=0.01, 95%CI (-0.96, 0.98), p=0.980; GAD-7 b=0.42, 95%CI(-0.43, 1.27), p=0.33; **Figure 3]**. This was consistent in the control group (**Figure 3**). There was no association between mental health outcomes and cumulative head impacts.

**Figure 4:**
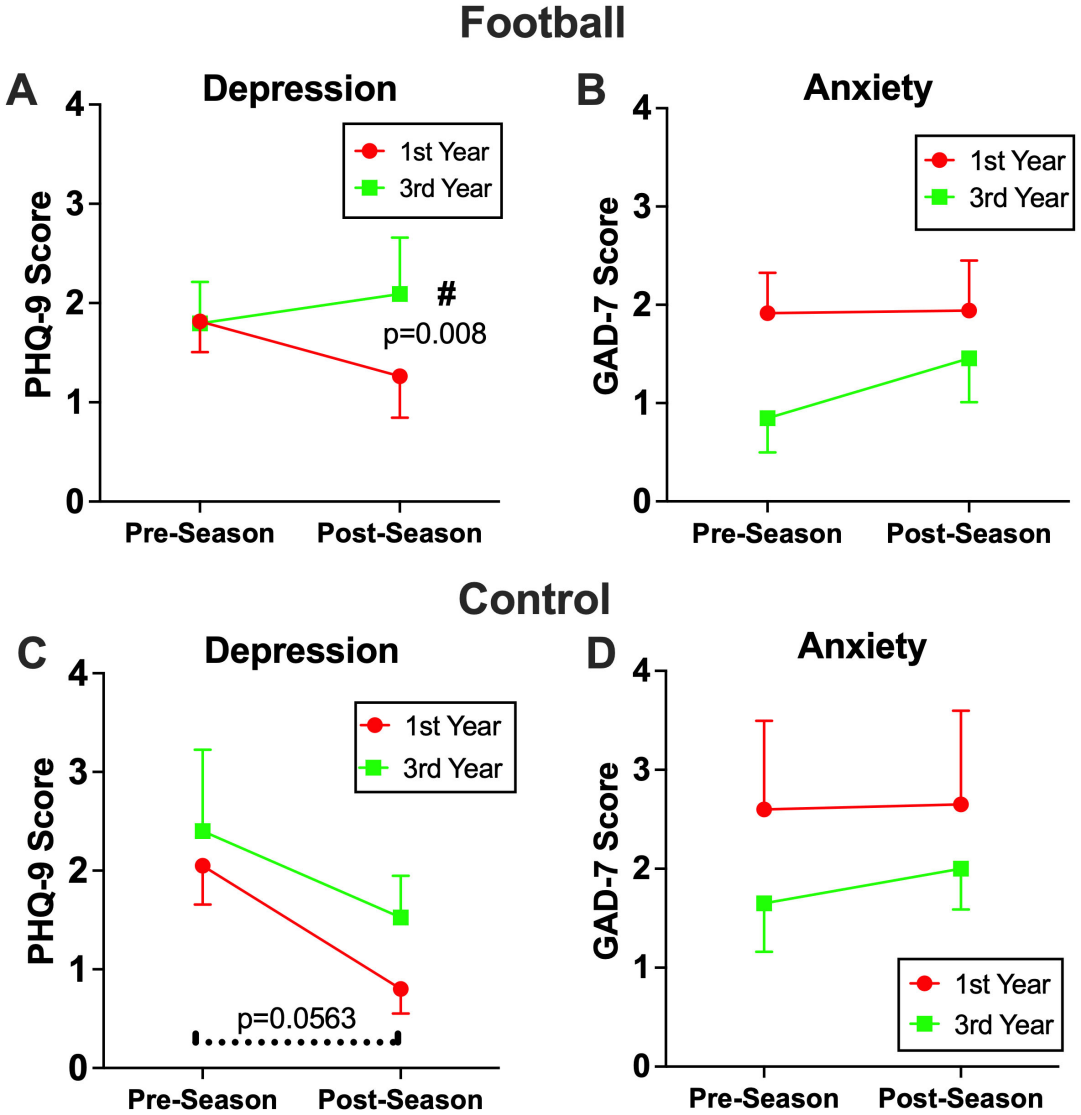
The effects of three consecutive season on mental health. Among football players who participated three consecutive seasons, significantly higher levels of depression symptoms **(A)**, but not anxiety **(B)**, were observed after their 3^rd^ season compared to their 1^st^ season (p=0.0148), which resulted in a significant group by time interaction (p=0.033). The control group exhibited decrease in depression symptoms in both their 1^st^ and 3^rd^ season **(C)**, while anxiety symptoms remained consistent across both seasons remained consistent across both seasons **(D)**.

Conversely, among football players who participated in three consecutive seasons, PHQ-9 scores showed a modest but statistically significant season (pre vs. post) by year (1^st^ vs. 3^rd^) interaction [PHQ-9: b=1.50, 95%CI (0.32, 2.66), p=0.015], reflecting higher postseason depressive symptom scores in the 3^rd^ year compared with the 1^st^ year. *Post-hoc* analysis also revealed that PHQ-9 scores at 3^rd^ year postseason were significantly greater than those of the 1^st^ year postseason [b=2.511, 95%CI (0.55, 4.47), p=0.008; **Figure 4]**. While control athletes did result in a significant decline in PHQ-9 scores from preseason to postseason after their first year of participation [b=-1.25, 95% CI (-2.53, 0.03), p=0.0563], score trends remained consistent across 1^st^ and 3^rd^ year of participation (**Figure 4**). Unlike PHQ-9, there were no significant multi-year effects in GAD-7 scores across seasons or between groups. There was no association between mental health outcomes and cumulative head impacts.

## Discussion

To our knowledge, this multisite, longitudinal study is the first to examine the relationship between RHI exposure during a high school football career and changes in mental health outcomes. The key findings were twofold: (1) consistent with our hypothesis and previous pilot study (18), a single season of football did not significantly influence depressive and anxiety symptoms compared to non-contact controls, even among players with frequent exposure to RHI; and (2) this stability in mental health persisted after two seasons in both the football and control groups; however, depressive symptoms after the third consecutive football season were significantly worse than postseason scores of their first season. This trend was absent in the control group. In line with our hypotheses, these findings suggest short-term football participation may not harm mental health, regardless of impact frequency, but prolonged exposure across seasons may contribute to cumulative psychological burden in adolescent players.

The novelty of the study findings is the emergence of depressive symptoms among adolescent football players after three consecutive seasons of participation, aligning with the notion that the effects of RHI may accumulate slowly and contribute to the progressive disturbances in mental health. For example, Guskiewicz et al. (26) conducted a cross-sectional survey study of 2, 552 retired professional football players (mean, 53.8 years) and identified elevated risks of depression diagnosis, which was modulated by lifetime concussion history, with 1 to 2 and 3 or more concussions increased likelihood of depression diagnosis by 1.5 and 3 folds, respectively. These findings were corroborated in former elite and amateur rugby players (27), former college (28), former professional (29), and active professional football players (30). In more acute settings, concussions can heighten depressive and anxiety symptoms, often manifested in a form of persistent post-concussive symptoms (PPCS) (31, 32). Upwards of 50% of adolescents experience PPCS even after 4 weeks post-concussion (31, 33), whereas 10-20% of college-aged adults and 15-30% of children have been shown to develop PPCS (34). Specifically, preteen athletes (8–12 yo) returned to sports faster (11.6 vs. 25.1 days) than teenage athletes (13–18 yo) (35), with teenagers facing 3 times higher risk of developing PPCS than preteens (36). Furthermore, teenagers who sustain concussive injuries have a 3-fold increase in the risk of developing depression and attention disorder within 5 years of injury (13). These data support that adolescence is a critical developmental window with heightened vulnerability to brain trauma. It is important to note that only a small fraction of football or control athletes (<7%) exceeded clinical thresholds (score >10) from preseason to postseason for all 3 years, suggesting that while statistically significant, most symptom fluctuations were not clinically significant.

In addition to concussive blows, researchers have begun warning the effects of subconcussive RHI in mental health. Montenigro et al. (15) proposed a threshold for lifetime RHI that can trigger mental and cognitive declines, with surpassing 1, 800 cumulative RHI being associated with a linear increase in depression risks. In seminal 2023 paper, McKee et al. (11), for the first time, illustrated that athletes exposed to RHI can develop CTE even below the age of 30. Based on informants’ data, most individuals diagnosed with CTE post-mortem exhibit increased depressive symptoms, impulsivity, and aggression before their passing (10, 11), emphasizing the importance of identifying progressive clinical symptoms early antemortem. Yet, opposing lines of research pose that the interaction between RHI and declines in mental health requires years of exposure beyond high school sports career. For example, both Iverson et al. (37) and Bohr et al. (38) found no association between contact sport participation up to high school and an increased risk of adverse mental health outcomes, including depression, suicide attempts, or cognitive decline. However, critical limitations prevail in both viewpoints surrounding contact sports participation and mental health wellbeing, where most data derived from online survey or informants’ reports in a cross-sectional design. Our data derived from one of the largest longitudinal studies in adolescent football players amalgamate previous perspectives and suggesting that high school football in short-term does not negatively impact mental health status (18), yet they may experience compounding effects over multiple seasons. While depressive symptoms appeared to increase after three consecutive football seasons, this association should be interpreted with caution. Given the observational nature of the study, causal inference cannot be established. Multiple contextual and developmental factors may also contribute to the observed pattern, including academic stress, social pressures, coaching or team changes, non-concussive injuries, and sleep disruption during adolescence. Therefore, these mental health declines in football players may have implications in their social and academic domains considering the complexity of brain development and societal demands during adolescence. Since adolescence is characterized by developmental increases in stress sensitivity and emotional fluctuation, and given evidence of generational rises in adolescent depressive and anxiety symptoms even among youth without head impact exposure (39), the control group in our study provides an essential benchmark for interpreting mental health trajectories independent of head impact exposure. Furthermore, a longitudinal survey study indicates no negative mental health implications of playing football during adolescence. However, high school football players exhibiting mental health issues were associated with an increased risk for depression diagnosis and suicidal ideation more than 20 years after their high school football career (17).

Contrary to depression, the lack of significant multi-year effects on anxiety symptoms is noteworthy. Factors such as a structured team environment, including social support from teammates and coaches, may mitigate feelings of being anxious (40). The prevalence of anxiety in adolescent athletic cohorts may be less prominent compared to depression. For example, among 471 adolescent soccer players, while mild to moderate depression were identified in 33 players (7.6%), the GAD-7 score indicated an anxiety disorder in 6 (1.4%) players. Compared to the general population, the prevalence of anxiety disorders was significantly lower in soccer players (41). Further research is warranted to clarify the differential impact of sports-related RHI on various aspects of mental health.

### Limitations

There are several limitations to this study. First, the cohort consisted exclusively of male athletes, which limits the generalizability to female athletes, whose responses may be moderated by anatomical, hormonal, and biomechanical differences. Second, although the sample size was robust, most participants were White and Non-Hispanic/Latino. Broader inclusion of racially and ethnically diverse individuals would enhance the generalizability of the findings. Third, concussion history, prior mental health diagnoses, and survey responses (PHQ-9 and GAD-7) were self-reported, which may introduce bias. However, the significant association between previous diagnoses and elevated scores supports the validity of the survey instruments. Fourth, the subgroup of athletes who completed three consecutive seasons was relatively small (39 football players and 20 controls), which limits statistical power to detect small changes in PHQ-9 scores and results in wide confidence intervals around our estimates. Thus, the observed increase in depressive symptoms after three seasons should be considered hypothesis-generating and preliminary. In addition, we conducted multiple mixed effects models across two correlated outcomes (PHQ-9 and GAD-7) and several time frames, which increases the risk of Type-I error. We therefore suggest appropriate caution when interpreting the three-season PHQ-9 findings, which requires replication in future studies. Lastly, a group of non-athlete controls may help untangled the potential mental health benefits of physical activity.

## Conclusions

Our data support that the participation in a single football season was not associated with changes in depressive or anxiety symptoms. However, among athletes with three consecutive seasons of participation, depression symptoms were heightened after their 3^rd^ season, compared to their 1^st^ or 2^nd^ season, suggesting a cumulative effect of RHI over time. The absence of similar changes in the control group reinforces the specificity of these effects to football-related participation. These findings underscore the importance of monitoring mental health across multiple seasons, as short-term assessments may not capture the gradual neuropsychological toll of continued exposure to subconcussive impacts.

## Data Availability

The raw data supporting the conclusions of this article will be made available by the authors, without undue reservation. Requests to access the datasets should be directed to the corresponding author.
