# Supplementary material for: Mental health trajectory throughout high school football career: a four-year prospective cohort study
Source: Front Psychiatry. 2025 Dec 3;16:1723687. doi: 10.3389/fpsyt.2025.1723687 (PMC12709013; doi:10.3389/fpsyt.2025.1723687)
Supplement: Supplementary file 1 [file Table1.docx]

**SUPPLEMENTAL TABLE**

| **Supplemental Table 1:** Average Mental Health Scores by Groups | | | | | | | |
| --- | --- | --- | --- | --- | --- | --- | --- |
|  | | **Football** | | | **Control** | | |
|  |  | **PHQ-9** | **GAD-7** | **PHQ-9** | | **GAD-7** |  |
| **1-year of participation** | Pre | 2.9 ± 3.9 | 2.4 ± 3.2 | 2.6 ± 2.8 | | 3.0 ± 4.1 |  |
|  | Post | 2.7 ± 4.1 | 2.3 ± 3.5 | 2.6 ± 3.4 | | 2.9 ± 3.7 |  |
| **2-years of participation** | Pre | 2.4 ± 3.1 | 2.2 ± 3.2 | 2.1 ± 2.7 | | 2.2 ± 3.2 |  |
|  | Post | 2.6 ± 3.9 | 2.5 ± 3.9 | 2.1 ±2.6 | | 2.0 ±3.1 |  |
| **3-years of participation** | Pre | 1.8 ± 2.6 | 0.8 ± 2.2 | 2.4 ± 3.7 | | 1.6 ± 2.2 |  |
|  | Post | 2.1 ±3.3 | 1.5 ± 2.6 | 1.5 ± 1.8 | | 2.0 ± 1.8 |  |
| **4-years of participation** | Pre | 3.4 ± 3.8 | 3.5 ± 4.6 | - | | - |  |
|  | Post | 2.3 ± 4.1 | 2.7 ± 3.7 | - | | - |  |
| Note: PHQ-9, Patient Health Questionnaire-9. GAD-7, General Anxiety Disorder-7. | | | | | | |  |
